# Genome-Wide Pathway Association Studies of Multiple Correlated Quantitative Phenotypes Using Principle Component Analyses

**DOI:** 10.1371/journal.pone.0053320

**Published:** 2012-12-28

**Authors:** Feng Zhang, Xiong Guo, Shixun Wu, Jing Han, Yongjun Liu, Hui Shen, Hong-Wen Deng

**Affiliations:** 1 Key Laboratory of Environment and Gene Related Diseases of Ministry Education, Faculty of Public Health, College of Medicine, Xi'an Jiaotong University, Xi'an, Shaanxi, China; 2 Department of Biostatistics, School of Public Health and Tropical Medicine, Tulane University, New Orleans, Louisiana, United States of America; University of Texas School of Public Health, United States of America

## Abstract

Genome-wide pathway association studies provide novel insight into the biological mechanism underlying complex diseases. Current pathway association studies primarily focus on single important disease phenotype, which is sometimes insufficient to characterize the clinical manifestations of complex diseases. We present a multi-phenotypes pathway association study(MPPAS) approach using principle component analysis(PCA). In our approach, PCA is first applied to multiple correlated quantitative phenotypes for extracting a set of orthogonal phenotypic components. The extracted phenotypic components are then used for pathway association analysis instead of original quantitative phenotypes. Four statistics were proposed for PCA-based MPPAS in this study. Simulations using the real data from the HapMap project were conducted to evaluate the power and type I error rates of PCA-based MPPAS under various scenarios considering sample sizes, additive and interactive genetic effects. A real genome-wide association study data set of bone mineral density (BMD) at hip and spine were also analyzed by PCA-based MPPAS. Simulation studies illustrated the performance of PCA-based MPPAS for identifying the causal pathways underlying complex diseases. Genome-wide MPPAS of BMD detected associations between BMD and KENNY_CTNNB1_TARGETS_UP as well as LONGEVITYPATHWAY pathways in this study. We aim to provide a applicable MPPAS approach, which may help to gain deep understanding the potential biological mechanism of association results for complex diseases.

## Introduction

Genome-wide association studies(GWAS) are successful for identifying common genetic variation underlying complex diseases in recent years [1]. In spite of the great power of GWAS, it may miss the causal genes with moderate genetic effects due to the stringent significant threshold of GWAS [2]. Moreover, the clinical manifestations of complex diseases usually arise from the interplay of multiple genetic and environmental risk factors through epigenetic and dynamic mechanism. Single gene can also participate in various biological processes. Identifying a small number of significant genes in GWAS may be insufficient to delineate the pathogenesis of complex diseases [3]. It is increasing recognized that a joint test of association between complex diseases and a group of functionally related genes, may provide more useful biological interpretations of association results [4], [5].

Motivated by the gene set enrichment analyses of microarray data [6], researchers proposed pathway association study approaches, which detected associations between complex diseases and a group of genes within a defined gene ontology or biological pathways [2]. Compared with SNP association studies, pathway association studies combine the association evidence of multiple functionally related genes, and potentially have greater power for revealing the biological mechanism underlying complex diseases [2]. For instance, a causal pathway with genes individually having weak genetic effects, but jointly contributing greatly to disease risks, is more likely to be detected at pathway level than at SNP level. Various pathway association study approaches were developed [7], [8], [9], [10], [11], [12], and successfully applied to genetic studies of complex diseases, such as osteoporosis and coronary heart disease [13], [14].

Current pathway association studies primarily focus on single important phenotype of complex diseases. A potential limitation of single phenotype pathway association studies is that single phenotype is sometimes insufficient to characterize complex diseases due to its complicated clinical manifestations. For example, obesity can be measured by body mass index, fat mass and proportions of fat mass in total body mass in practice. To address this issue, some researchers collected a set of disease-related phenotypes, and conducted pathway association tests of each phenotype ignoring the correlation among multiple disease phenotypes [15]. Given the difference of genetic structure underlying different disease phenotypes, it may be difficult to get replicated associations among different single phenotype pathway association studies. Additionally, multiple testing corrections were usually requested to ensure normal type I error rates in these studies. Because of the correlation among multiple disease phenotypes, multiple testing corrections (for example Bonferroni), may be too strict to miss the pathways with moderate association signals.

Recently, multivariable analyses approaches were applied to SNP association studies, which could simultaneously detect associations between SNPs and multiple disease phenotypes [16], [17]. The causal genes with moderate association signals in single phenotype SNP association studies are likely to present strong association signals in multiple phenotypes SNP association studies avoiding multiple testing corrections. It may be reasonable to consider that combining the genetic information of multiple disease phenotypes was potentially able to enhance the association signals of causal pathways, and therefore increased the power of pathway association studies of complex diseases. However, to the best of our knowledge, few multiple phenotypes pathway association study(MPPAS) approach is available now.

In this study, we present a flexible MPPAS approach using principle component analyses (PCA). In our approach, PCA is first applied to multiple correlated quantitative phenotypes for exacting a set of orthogonal phenotypic components. The extracted phenotypic components are then included into pathway association analyses instead of original disease phenotypes. Four statistics combining the association evidence of multiple genes within testing pathways, were proposed for assessing the overall association strength of the pathways with target traits. To illustrate the application of our method, extensive simulation studies using the real data from the HapMap project, were conducted to evaluate the power and type I error rates of PCA-based MPPAS under various scenarios, considering sample sizes, additive and interactive genetic effects. PCA-based MPPAS can be applied to GWAS data. A real GWAS data set of osteoporosis was analyzed by PCA-based MPPAS in this study.

## Results

### Simulations

**Figure 1:**
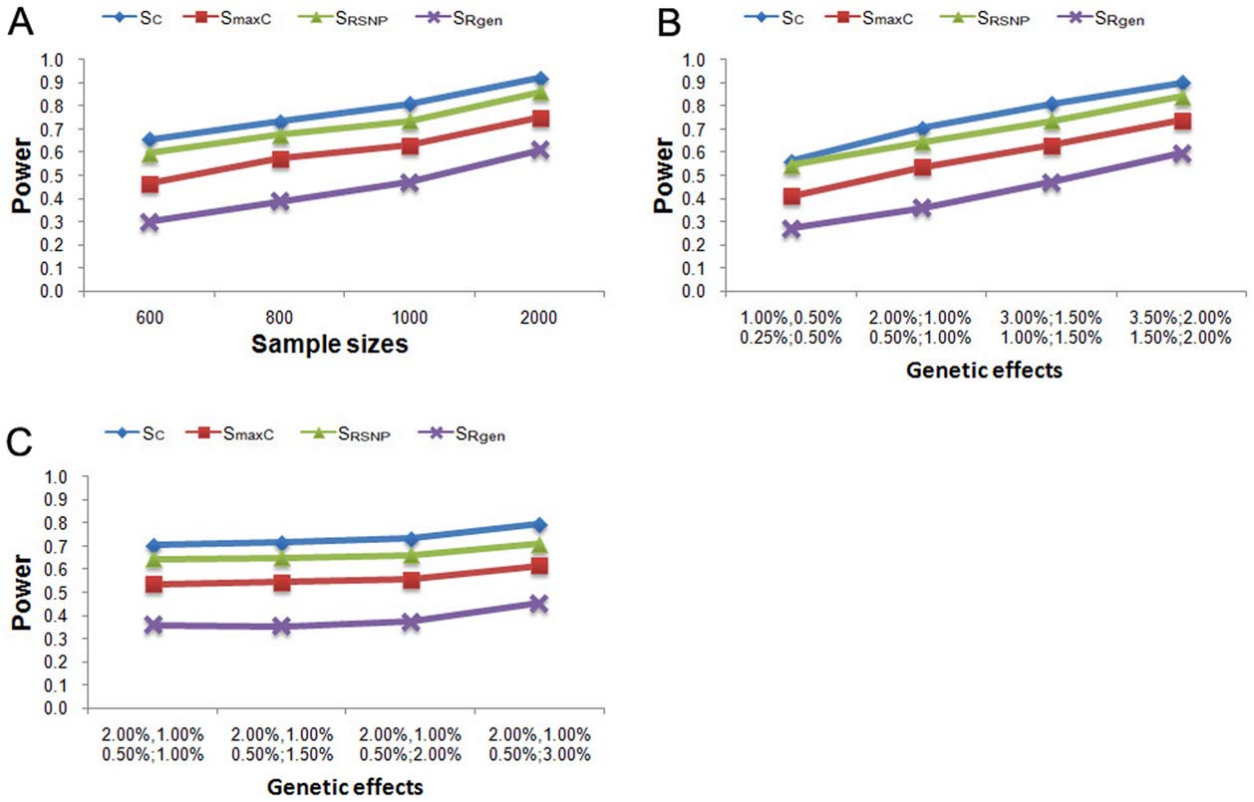
Power simulating results of PCA-based MPPAS using 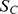, 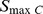, 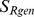 and 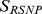 statistics under various sample sizes (A) and genetic effects(B&C).

The power of PCA-based MPPAS using 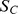, 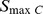, 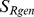 and 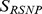 statistics, were evaluated by the simulation studies considering sample sizes, additive and interactive genetic effects. Figure 1A presents the power comparison results of 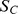, 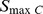, 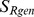 and 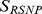 under various sample sizes. As expect, the power of PCA-based MPPAS trended to increase with increasing sample sizes in this study. 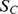 performed better than other statistics, and attained the highest power 92.07% with 2000 samples. 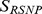 performed slightly worse than 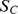, but outperformed 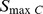 and 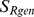.

Figure 1B summarizes the power comparison results of 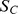, 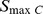, 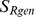 and 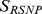 under various genetic effects. We observed significant impact of genetic effects on the performance of PCA-based MPPAS. The power of 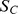, 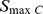, 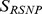 and 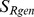 increased with increased genetic effects of causal pathways in this studies. Consistent with the simulation results of sample sizes, 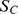 attained the highest power, following by 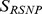, 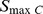 and 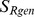 under various genetic effects investigated by this study. The simulation results of interactive genetic effects are presented in Figure 1C. We observed increased power of PCA-based MPPAS as the interactive genetic effects of causla pathways increasing. 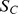 outperformed 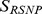, 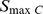 and 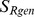 under various interactive genetic effects investigated by this study.

**Figure 2:**
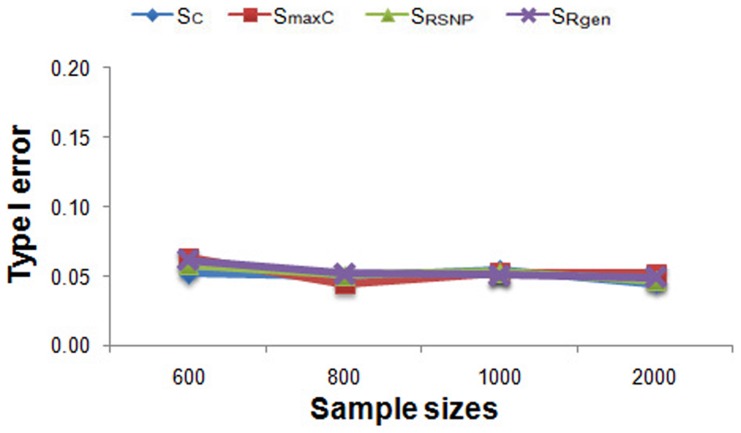
Type I error rate simulating results of PCA-based MPPAS using 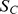, 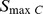, 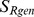 and 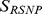 statistics under various sample sizes.

Figure 2 plot the type I error rates of PCA-based MPPAS using 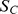, 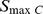, 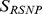 and 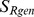 for testing association under various sample sizes. The type I error rates of 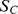, 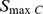, 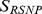 and 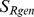 are not significant different from normal level (α = 0.05) under various simulating parameters investigated by this study.

### Genome-wide MPPAS of BMD

**Figure 3:**
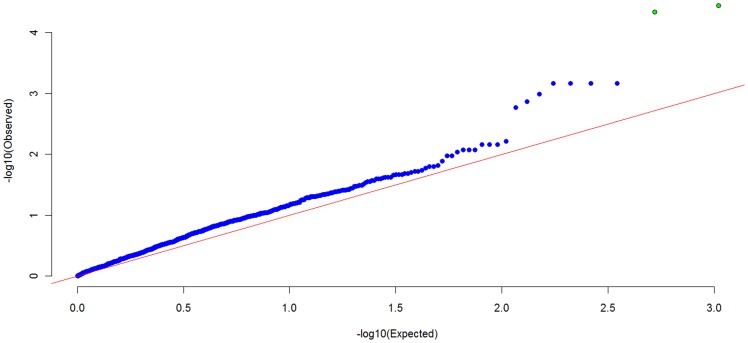
Q-Q plot of genome-wide MPPAS results of BMD at spine and hip.

**Figure 4:**
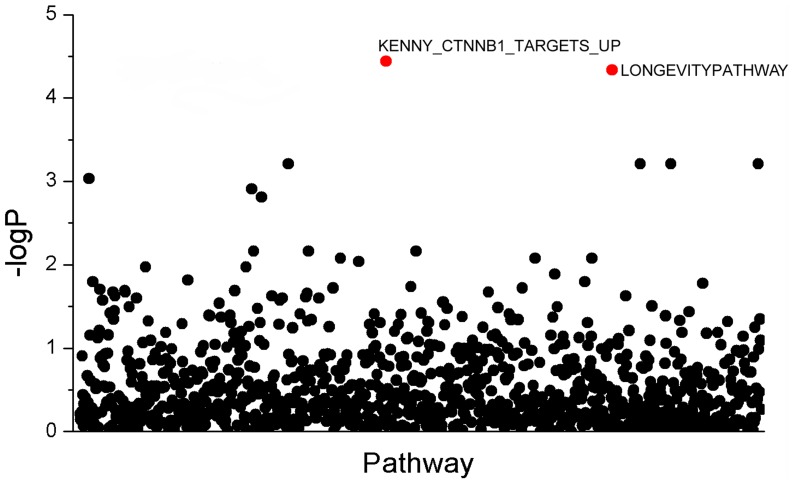
Plot of genome-wide MPPAS results of BMD. The significant pathways are highlighted in red. Significant pathways were defined by *p* values≤5.19×10^−5^ after Bonferroni correction(0.05/963).

Figure 3 and figure 4 summarizes the genome-wide MPPAS results of BMD at spine and hip. With PCA-based MPPAS using 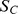, we identified 2 pathways associated with BMD, including KENNY_CTNNB1_TARGETS_UP(*p* = 4.62×10^−5^) and LONGEVITYPATHWAY (*p* = 3.59×10^−5^). Detailed description of KENNY_CTNNB1_TARGETS_UP and LONGEVITYPATHWAY pathways can be found at GSEA Molecular Signatures Database (http://www.broadinstitute.org).

## Discussion

Pathway association studies are based on the fact that different causal genes of a complex disease are likely to be functionally related, for instance belonging to same biological pathways [18]. Therefore, examining the overall association strength of a pathway may provide improved power for pathogenetic studies of complex diseases, especially for the pathways with each gene having small phenotypic effects, but all genes jointly contributing greatly to disease risks. However, current pathway association studies primarily focus on single important phenotype of complex diseases, which may miss the pathways with weak genetic effects. In this study, we presented a simple PCA-based MPPAS approaches, which can simultaneously test multiple correlated quantitative phenotypes. Simulations were conducted to evaluate the performance of PCA-based MPPAS using 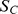, 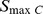, 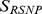 or 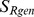, and illustrated the application of PCA-based MPPAS for pathway association studies of complex diseases. We also observed significant impact of sample sizes and genetic effects on the performance of PCA-based MPPAS. PCA-based MPPAS using 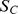 statistic appeared to outperform PCA-based MPPAS using 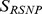, 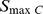 or 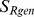 statistics in this study.

The PCA-based MPPAS have potentially two advantages over single phenotype pathway association studies. First, single phenotype is sometimes insufficient for characterizing complex diseases. In this situation, one strategy is to collect multiple disease phenotypes, and conduct single phenotype pathway association studies following by picking up the shared pathways with significant association signals among different studies. One issue of this approach is that the association finding of common causal pathways may be difficult to be replicated across various single phenotype pathway association studies, due to the difference of mechanism underlying different disease phenotypes. Second, multiple testing corrections are usually requested by this approach. Because of the stringent significant threshold after multiple testing corrections, the causal pathways with moderate but meaningful associations may be missed by single phenotype association studies. In contrast, MPPAS incorporate the genetic information of multiple correlated disease phenotypes into single test statistic. The causal pathways with moderate association signals in single phenotype pathway association studies, are likely to present strong association signals in MPPAS avoiding multiple testing corrections.

PCA-based MPPAS can be applied to GWAS data. A real GWAS data of BMD was used to assess the performance of PCA-based MPPAS in this study. We observed significant associations with BMD for KENNY_CTNNB1_TARGETS_UP and LONGEVITYPATHWAY pathways. Previous studies may provide some hints for understanding the associations detected by this study. For instance, previous studies found that the GHR, GH1, ATK1, IGF1 and IGF1R genes of LONGEVITYPATHWAY (containing 14 genes) contributed to the variation of BMD [19], [20], [21], [22]. KENNY_CTNNB1_TARGETS_UP consists of a set of genes being the target of Wnt pathway, which plays an important in the regulation of bone mass accrual [23], [24]. Further studies may be needed to validate the associations detected by this study.

A potential extension of our approach is that haplotype association studies may also be applied to PCA-based MPPAS instead of SNP association studies used by this study. It is known that haplotype association studies preserving the polymorphism and linkage disequilibrium information of multiple adjacent SNPs, was more powerful for detecting rare genetic variants than SNP association studies in some cases [25], [26]. For instance, the causal genes with multiple SNPs jointly having significant phenotypic effects, but individual SNP making a small contribution, is likely to be missed by SNP association studies. PCA-based MPPAS using haplotype as basic unit for association testing, may provide additional information for reveal the biological mechanism of complex diseases. Further studies may be worth to investigate the performance of MPPAS using haplotype as association testing unit.

Population stratification is a problem in population–based SNP association studies. SNP association studies conducted in an admixed population with subpopulations having different allele frequency distribution, may result in spurious association results [27]. Because most of current pathway association studies are based on the results of SNP association studies, the performance of pathway association studies may also suffer from the impact of population stratification. The best solution is to collect genetic unrelated subjects as study samples. Additionally, some statistical methods can also be applied to SNP association studies for correcting population stratification, such as Structure and Eigensoft [28], [29]. Linkage disequilibrium(LD) is another concern with pathway association studies, which may result in extensive spurious associations [7]. In this study, the significance levels of testing statistics of PCA-based MPPAS were evaluated by Monte Carlo permutations, which used the same individuals and maintained the same LD structure between original datasets and subsequent randomized datasets. PCA-based MPPAS do not depend on specific statistical assumption, for example the normality assumption of target traits. This approach minimizes the impact of LD on the performance of PCA-based MPPAS. The computational cost of PCA-based MPPAS is also acceptable in practice. For instance, our genome-wide PCA-based MPPAS of BMD needed about 12 days(1000 subjects and 50,000 replicates).

In summary, we present a flexible PCA-based MPPAS approach avoid multiple testing corrections. Simulations and real GWAS data analyses results illustrated the application of PCA-based MPPAS for identifying causal pathways underlying complex diseases. PCA-based MPPAS may help to overcome the limitations of single phenotype pathway association studies, and gain deep understanding the molecular mechanism of association results for complex diseases.

## Materials and Methods

### Ethics Statement

All studies were approved by the Institutional Review Boards of Xi'an Jiaotong University. Informed-consent documents were read and signed by all study participants.

### General Model

Suppose a sample of *n* unrelated subjects and k quantitative phenotypes, which was determined by a biological pathway with *m* genotyped SNPs. Let 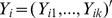 denote the *k*×1 phenotype vector, and 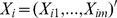 denote the *m*×1 genotype vector of subject *i* (*i* = 1,…,*n*). In this study, we coded 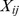
(*j* = 1,…,m) to be 0, 1 or 2, representing the copy number of minor allele of subject *i* at the *jth* SNP. 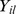
(*l* = 1,…,*k*) can be formulated as
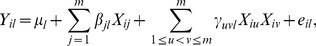
(1)where 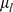 denotes the mean of the l*th* quantitative phenotypes. 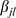 denotes the additive genetic effect of SNP *j* for the *lth* quantitative phenotypes. 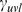 denotes the interactive genetic effect between SNP *u* and SNP *v* for the *lth* quantitative phenotypes. 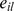 denotes the residual environmental effect of subject *i* for the *lth* quantitative phenotype.

### Extracting Phenotypic Components by PCA

Because different phenotypes may be measured using different units in practice, we first standardize the original quantitative phenotypes. Let 
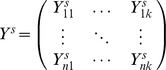
 denotes the matrix of *k* standardized quantitative phenotypes for *n* subjects. The matrix element 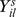 can be computed by
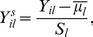
(2)where 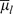 and 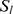 denote the mean and standard deviation of the *lth* quantitative phenotype, respectively.

PCA(implemented by R software, http://www.r-project.org/) is then applied to 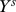 for extracting k orthogonal phenotypic components. Following standard PCA precedure, let 
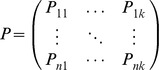
 denotes the matrix of *k* extracted phenotypic components for *n* subjects. The matrix element 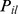 is calculated by
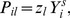
(3)where 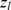 is calculated by PCA and denotes the eigenvector of the *lth* phenotypic components. 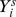 denotes the standardized phenotypic vector of subject *i*.

### Pathway Association Testing Statistics

The phenotypic components extracted by PCA are included into pathway association analysis instead of original quantitative phenotypes. Suppose a pathway with *r* genes and *m* genotyped SNPs. For a given gene within the pathway, we first detect associations between each SNP of the gene and each phenotypic component. For each gene, the largest statistic of all SNPs mapped to the gene is assigned to the gene as the statistic of the gene [7]. Let 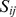
(*i* = 1,…,*k* and *j* = 1,…*r*) denotes the largest statstic of gene *j* for the *ith* phenotypic component. Let 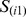
≥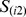 ≥…≥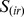 denote the ordered statstics of the pathway for the *ith* phenotypic component. Based on the idea that a pathway with more genes associated with target traits, is more likely to be disease-causing pathway, we present four statistics to evaluate the overall association strength of a pathway with target traits. The first one takes a linear combination of statistics of all genes within the pathway, defined by
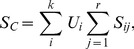
(4)where 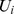 is computed by PCA and denotes the proportion of phenotypic variance explained by the *ith* phenotypic component. The phenotypic information harboring by different components are different, and can be measured by the explained proportions of phenotypic variation in PCA. 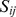 are weighted by the explained proportion of phenotypic variance, which gives higher weight to the phenotypic components explained larger part of variance of original *k* quantitative phenotypes.

Because of combining the association evidence of all genes within the pathway, 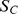 may be susceptive to the impact of pathway sizes. Consider an extreme case that we have a vary large pathway with only one significant gene. In this situation, the true association signal of causal gene may be masked by the nosie of other genes within the pathway. Therefore, we proposed the second statistic 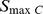, which taked the maximum value of averaged statistics within the pathway. 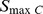 is defined by
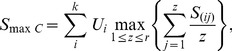
(5)where 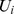 is defined in equation 4.

Recently, SNP raio tests were proposed for pathway association studies [10]. This approach compared the ratio of significant and no-significant SNPs within a pathway to the distribution of ratios derived from GWAS results of randomized phenotypes. The pathways with larger part of genes or SNPs associated with disease phenotypes is more likely to contribute to disease risks. In this study, we extended the ratio tests to PCA-based MPPAS, and considered two ratio testing approaches, SNP ratio tests and gene ratio tests. Let 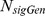 and 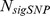 denote the numbers of significant genes and SNPs within testing pathways. The pathway ratio testing statistics can be expressed as
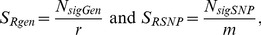
(6)where *r* and *m* denotes respectively the numbers of genes and SNPs within the pathway.

For statistical tests, a permutation precedure was implemented to evaluate the significance levels of 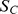, 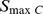, 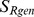 and 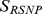 in this study. During each permutation, the sample labels were randomly assigned to individuals following by computation of 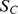, 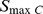, 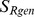 and 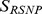, respectively. 2,000 Monte Carlo permutations were conducted to obtain the empirical distributions of 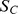, 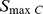, 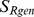 and 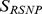. The significance levels of 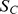, 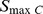, 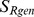 and 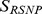 were finally evaluated according to the obtained empirical distributions.

### Simulations

#### Genotype simulation

HAPGEN program was used here for genotype simulations [30], [31]. Based on known haplotype data, HAPGEN can simulate whole-genome genotype data by implementing a hidden Markov model [30], [31]. Specific for this study, the genome-wide haplotype data, minor allele frequencies (MAF) and D′ of Caucasian were downloaded from the HapMap website(http://hapmap.ncbi.nlm.nih.gov/downloads/index.html.en). HAPGEN was then used to simulate genome-wide genotype data of Caucasian with default running parameters recommended by HAPGEN developers.

333 pathways or gene ontology with sizes ranging from 20 to 40, were collected from four public pathway databases, including BioCarta(http://www.biocarta.com), KEGG(http://www.genome.jp/kegg/), Ambion GeneAssist Pathway Atlas(http://www5.appliedbiosystems.com/tools/pathway/), and GSEA Molecular Signatures Database(http://www.broadinstitute.org). The obtained pathway-gene annotation file was used to link pathway and gene information in following pathway simulation studies.

#### Phenotype simulations

Genetic epistatic model was applied here for quantitative phenotype simulations. Suppose a complex disease underlying by a biological pathway, was mesured by three correlated quantitaitve phenotypes, Q_1_, Q_2_ and Q_3_. During each phenotype simulation, we first randomly selected a pathway as the causal pathway. Three SNPs (SNP_1_, SNP_2_ and SNP_3_) were then randomly selected from different genes of the causal pathway as the causal loci of Q_1_. The same precedure was also conducted for Q_2_ and Q_3_, respectively. Let 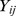 denotes the *jth* quantitative phenotype value of subject *i*, defined by
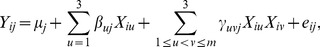
(7)where 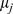 denotes the mean of the *jth* quantitative phenotype. 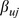 denotes the additive genetic effect of SNP *u* for the *jth* quantitative phenotype. 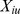
(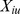 = 0, 1 or 2) denotes the copy number of minor allele of subject *i* at SNP *u*. 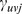 denotes the interactive genetic effect between SNP *u* and SNP *v* for the *jth* quantitative phenotype. Without loss of generality, we assume that there was an interactive genetic effect between SNP_1_ and SNP_3_ for Q_1_ in this study. 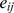 denotes the residual environmental effect of subject *i* for the *jth* quantitative phenotype, and follow a zero-mean normal distribution with variance 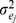.

#### Data analysis

The simulated genotype and phenotype data were simultaneously analyzed by PCA-based MPPAS using 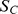, 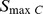, 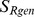 and 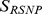, respectively. Sample sizes, additive and interactive genetic effects were controlled to simulate various scenarios of pathway association studies in practice. Detailed parameter designs are presented in Table 1. 1,000 replicates were conducted for each parameter setting. Power and type I error rates were calculated respectively as the proportions of positive association results (*p* values≤0.05) obtained from the pathways simulated with and without genetic effects in 1,000 replicates. All our data simulations and analyses were implemented with statistical package R [32], except for SNP association tests implemented by PLINK [33].

**Table 1:** Parameter configurations in the simulation studies.

	Sample size	Genetic effect^a^			
		SNP_1_	SNP_2_	SNP_3_	SNP_1_×SNP_3_
Simulation 1	**600**	3.00%	1.50%	1.00%	1.50%
	**800**	3.00%	1.50%	1.00%	1.50%
	**1000**	3.00%	1.50%	1.00%	1.50%
	**2000**	3.00%	1.50%	1.00%	1.50%
Simulation2	1000	**1.00%**	**0.50%**	**0.25%**	**0.50%**
	1000	**2.00%**	**1.00%**	**0.50%**	**1.00%**
	1000	**3.00%**	**1.50%**	**1.00%**	**1.50%**
	1000	**3.50%**	**2.00%**	**1.50%**	**2.00%**
Simulation3	1000	**2.00%**	**1.00%**	**0.50%**	**1.00%**
	1000	**2.00%**	**1.00%**	**0.50%**	**1.50%**
	1000	**2.00%**	**1.00%**	**0.50%**	**2.00%**
	1000	**2.00%**	**1.00%**	**0.50%**	**3.00%**

a denote the phenotypic variance explained by the additive genetic effects of SNP_1_, SNP_2_ and SNP_3_ as well as an interactive effect between SNP_1_ and SNP_3_, respectively.

333 pathways with sizes varying from 20 to 40, were collected from public pathway databases and used for pathway simulations.

### Application to real GWAS Data of BMD

PCA-based MPPAS using 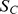 was applied to a real GWAS data consisting of 1,000 unrelated US whites. The sample characteristics and experimental design have been detailed in previous study [34]. Briefly, Affymetrix 500 k SNP arrays were used to genotype a total of 500,568 SNPs. After quality control, 312,172 SNPs covering 14,585 genes were retained for MPPAS of BMD in this study. Areal BMD of spine and hip were measured by dual-energy X-ray absorptiometry (DXA) with Hologic QDR 4500W densitometers (Hologic, Inc., Bedford, MA, USA). Age and sex were used to adjust the raw spine and hip BMD values as covariates for subsequent analyses. The adjusted BMD data were normally distributed. 963 pathways or gene ontology with sizes varying from 5 to 168, were derived from public pathway databases, including BioCarta, KEGG, Ambion GeneAssist Pathway Atlas, and GSEA Molecular Signatures Database. PCA-based MPPAS using 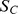 was used to detect association between each pathway and BMD. 50,000 replicates were conducted to evaluate the empirical *p* values of 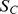 for each gene set investigated in this study. Significant pathway were defined by *p* values≤5.19×10^−5^ after Bonferroni correction(0.05/963).
